# Beliefs in “Brilliance” and Belonging Uncertainty in Male and Female STEM Students

**DOI:** 10.3389/fpsyg.2019.01114

**Published:** 2019-05-28

**Authors:** Anne Deiglmayr, Elsbeth Stern, Renate Schubert

**Affiliations:** ^1^ETH Zürich, Department of Humanities, Social and Political Sciences, Zurich, Switzerland; ^2^Faculty of Education, University of Leipzig, Leipzig, Germany

**Keywords:** field-specific ability beliefs, belonging uncertainty, STEM gender gap, gender stereotypes, university students

## Abstract

A wide-spread stereotype that influences women’s paths into STEM (or non-STEM) fields is the implicit association of science and mathematics with “male” and with requiring high levels of male-associated “brilliance.” Recent research on such “field-specific ability beliefs” has shown that a high emphasis on brilliance in a specific field goes along with a low share of female students among its graduates. A possible mediating mechanisms between cultural expectations and stereotypes on the one hand, and women’s underrepresentation in math-intensive STEM fields on the other hand, is that women may be more likely than men to feel that they do not belong in these fields. In the present study, we investigated field-specific ability beliefs as well as belonging uncertainty in a sample of *n* = 1294 male and female university students from five STEM fields (Mathematics, Physics, Computer Science, Electrical Engineering, and Mechanical Engineering) at a prestigious technical university in Switzerland. Field-specific ability beliefs of both men and women emphasized brilliance more in more math-intensive fields (Mathematics, Physics) than in less math-intensive fields (Engineering). Women showed higher beliefs in brilliance than men did, and also reported higher levels of belonging uncertainty. For both genders, there was a small, positive correlation (*r* = 0.19) of belief in brilliance and belonging uncertainty. A relatively small, but significant portion of the effect of gender on belonging uncertainty was mediated by women’s higher belief in brilliance.

## Introduction

Although the gender-gap in achievement in STEM fields has narrowed down in recent years, women remain underrepresented in many math-intensive fields (Ceci et al., 2014; Wang and Degol, 2017). The dimension of this gender gap and possible explanations for its sustained existence have been analyzed from many perspectives, and based on large data sets, in recent years (for overviews, see for example: Ceci et al., 2014; Miller et al., 2015; Cheryan et al., 2017; Wang and Degol, 2017; Stoet and Geary, 2018). Analysts generally agree that the underrepresentation of women in math-intensive STEM fields results from the interplay of multiple factors. Biological factors and differences in basic cognitive abilities may contribute to the phenomenon, but cannot explain the substantial cross-cultural and historic variability in gender inequality in entry into STEM (Berkowitz et al., in press; Wang and Degol, 2017; Stoet and Geary, 2018). There areindications that women face implicit negative biases when decision-makers judge their abilities and performance in math-intensive STEM fields, for instance when teachers grade girls (Hofer, 2015) or when faculty members rate applicants (Moss-Racusin et al., 2012). To a large extent, however, the underrepresentation of women in STEM fields seems to reflect choices that girls and women make themselves, e.g., by choosing hobbies, academic specializations, study subjects, or career paths leading them into less math-intensive or non-STEM fields (Ceci et al., 2014). Of course, even though such choices seem to be free at the first glance, they are constrained by cultural expectations and stereotypes that associate science and mathematics with stereotypically male, rather than stereotypically female traits (e.g., Thébaud and Charles, 2018).

A wide-spread stereotype that influences women’s paths into STEM (or non-STEM) fields is the implicit association of science and mathematics with “male” traits (Nosek et al., 2002). This stereotype is present even in societies with high levels of gender-equity (e.g., Miller et al., 2015). The association of science as being male is linked to gender-specific attributions of success: across a broad range of fields and age groups, success has been shown to be implicitly attributed to innate talent for males and to hard work for females (Proudfoot et al., 2015; Verniers and Martinot, 2015). Recent research on “field-specific ability beliefs” has shown that a high emphasis on “brilliance” (i.e., raw talent) as a requirement for success goes along with a low share of female students among the graduates of a specific field (Leslie et al., 2015; Meyer et al., 2015). To summarize, across academic fields, success is attributed more to some form of innate “brilliance” for males than females. In combination with the field-specific belief in the importance of “brilliance” that dominates many STEM fields, this may result in negative stereotypes against women.

A possible mediating mechanisms between cultural expectations and stereotypes on the one hand, and women’s underrepresentation in math-intensive STEM fields on the other hand, is that women may be more likely than men to feel that they do not belong in these fields. Social belonging, more precisely the feeling of “belonging uncertainty” (Walton and Cohen, 2007), has been linked to students’ persistence, well-being, and academic achievement in STEM subjects (Walton and Cohen, 2011; Walton et al., 2015). Belonging uncertainty, to specify the term, is an individual’s perception that “people like me do not belong here” (Walton and Cohen, 2007, p. 83). Often, belonging uncertainty is reported by members of underrepresented social groups against whom negative stereotypes exist, like women in math-intensive STEM fields (Walton et al., 2015), or minority students in college (Walton and Cohen, 2011). Students experiencing belonging uncertainty are more negatively affected by difficulties they face during their studies, and are more likely to give up their course of study or study field (Walton and Cohen, 2011; Walton et al., 2015).

We argue that the perception that a specific STEM field requires male-associated “brilliance” may contribute to women’s belonging uncertainty with regard to the respective field. Thus, it may contribute to women’s reluctance to choose such a field, or to remain in it when facing difficulties. We had the chance to correlate field-specific ability beliefs and belonging-uncertainty in a group of students from five different STEM subjects at a prestigious university in Central Europe. To our knowledge, this is the first study measuring field-specific ability beliefs (Leslie et al., 2015) in a sample of university students enrolled in the respective fields. The field-specific ability beliefs of faculty are of course important, as they may influence the reactions and feedbacks that male vs. female students receive for their efforts (Leslie et al., 2015). However, the field-specific ability beliefs held by the students themselves will likely have a more direct impact on their feeling of belonging, and will thus influence their willingness to choose and to persist in a math-intensive STEM field. We hypothesize that this is particularly true for female students: There are negative stereotypes against women regarding their possession of raw talent (“They might be the harder workers, but compared to men, women have lower levels of raw talent”; compare Proudfoot et al., 2015; Verniers and Martinot, 2015). These may lead to belonging uncertainty, and eventually underrepresentation of women, in fields that they perceive as requiring high levels of raw talent that cannot be compensated for by hard work.

## Study Goals

In the present study, we aim to investigate field-specific differences in ability beliefs in university students in a range of STEM subjects, and to demonstrate a correlation between field-specific ability beliefs emphasizing “brilliance” on the one hand and belonging uncertainty on the other hand.

A further goal is to replicate the findings by Leslie et al. (2015) in a student sample. The authors found a negative correlation between faculty’s endorsement of brilliance as a prerequisite for success and the percentage of female Ph.D. recipients in the respective field. Here, we explore whether there also is a negative correlation between students’ endorsement of brilliance as a prerequisite for success in their chosen field and the percentage of female students in the same field. Our sample allows, to some extent, to disentangle the impact of the degree of “math intensiveness” on the one hand, and the minority status of women on the other hand: We recruited participants from the departments of Mathematics, Physics, Computer Science, Electrical Engineering, and Mechanical Engineering. Traditionally, at least at our university, the demands on competencies in mathematics required by the curriculum are higher in the study programs in Mathematics and Physics than they are in the subjects of Computer Science, Electrical Engineering, and Mechanical Engineering. At the same time, the proportion of female students is lower in these three programs than in Mathematics and Physics. This allows us to explore whether belonging uncertainty and the endorsement of brilliance are, in our sample, lower in the subjects with the highest math intensiveness (Mathematics, Physics), or in the subjects with the lowest number of women (Computer Science, Electrical Engineering, and Mechanical Engineering).

The research questions and hypotheses of this study are:

(1) To what extent do the field-specific ability beliefs of university STEM students emphasize “brilliance”? Are there differences between the different STEM subjects and between genders? Is the highest endorsement of “brilliance” to be found in the very math-intensive fields, such as Math and Physics, or in study programs with the a very low percentage of female students, such as Engineering?
(2) To what extent can field-specific ability beliefs emphasizing “brilliance” predict belonging uncertainty in male and in female STEM students? Based on the theoretical framework behind the concept of belonging uncertainty, doubt about one’s competence (e.g., from experiencing actual failure, or from activated negative stereotypes about one’s social group) should be the more detrimental to one’s feeling of belonging, the more one believes that success depends on some form of innate talent or “brilliance.” Therefore, our hypotheses are that (a) there is a positive correlation between belief of brilliance and belonging uncertainty, and (b) that gender differences in belief in brilliance mediate gender differences in belonging uncertainty.

## Materials and Methods

### Sample

Our participants were first-year students enrolled at a prestigious, rather male-dominated, technical university in Switzerland, i.e., the ETH Zurich. They came from five departments (Mathematics, Physics, Computer Science, Electrical Engineering, and Mechanical Engineering), all representing math-intensive STEM fields, although to a different degree. The curricula in Mathematics and Physics have a much stronger requirement in mathematics than the Computer Science and Engineering curricula (cf. Berkowitz and Stern, 2018). On the other hand, the proportion of female students is typically higher in Mathematics and Physics than in Computer Science and Engineering. All first-year students in the five departments were invited to participate in a short online survey toward the middle of their first term. In two consecutive years (2016 and 2017 cohorts), the survey was sent out to the students by the University administration as part of a larger teaching development project. The data of both cohorts was combined for all analyses. Initially, roughly 3000 students were invited to participate in the survey. A total of *n* = 1424 participated, of which *n* = 1294 gave informed consent for their data to be used for research purposes. Data on the survey items relevant for this study were missing for *n* = 3 students, leaving a total sample of *n* = 1291. The sample included *n* = 235 women (18% of the sample), which is roughly representative for the mean male-to-female ratio in the five surveyed departments.

The data for calculating the percentage of female student’s in the five departments were collected from the University administration and encompass the entire cohorts of students starting their studies in the five departments in Fall 2016 or Fall 2017. The percentages were 25% female students in Mathematics, 19% in Physics, 15% in Electrical Engineering, and 12% in Mechanical Engineering and Computer Science, respectively.

### Survey

The online survey had several parts, of which only one is relevant for the study at hand. In a first part of the survey, students answered questions that concerned a change in the first-year examination mode (13 items). The second part of the survey assessed students’ fields-specific-ability beliefs and belonging uncertainty (7 items). The third part of the survey asked students for alternative plans to studying (4 items), and the fourth part aimed to gauge their general well-being at their new school (4 items). Finally, students could give feedback in an open-answer item. At the end of the survey, students received information on the further handling of their data and were asked to give their consent to use their answers for research purposes.

For the current study, only students’ answers in the second part of the survey are of relevance. In this part of the questionnaire, students’ answered the items of the field-specific ability belief scale (FSAB; 4 items) originally published by Leslie et al. (2015) and of the belonging uncertainty scale (BU; 3 items) by Walton and Cohen (2007). The items of both scales were presented on one page, intermixed in one block titled “Your studies at [school name].” All items were rated on a 7-point answering scale. Only the endpoints were labeled as “do not agree at all” (German original: “trifft gar nicht zu”; coded as 1) or “completely agree” (German original: “trifft völlig zu,” coded as 7).

The FSAB items were translated to German and reworded to assess the perception of non-faculty members, i.e., students in a given field (sample item: “Being successful in my subject of study requires a special aptitude that just can’t be learned.”). The BU items were translated to German and adapted to be specific for the students’ school, i.e., ETH Zurich (sample item: “When things are going badly, I feel that maybe I don’t belong at ETH after all.”). The complete list of questions (in German and in the English re-translation) is available as Supplementary Table 1.

## Results

### Field-Specific Ability Beliefs

In our sample, the internal consistency of the four FSAB items proved satisfactory (Cronbach’s α = 0.78). Thus, for all further analyses, the mean of all four items was calculated. Table 1 gives the mean of the FSAB scale for students of the five departments and for both genders. Higher values indicate greater endorsement of brilliance or innate talent as a prerequisite of success in the chosen field of study (the answering scale runs from 1 to 7, with 4 representing the middle of the scale). All but one of the mean values are in the lower half of the scale, indicating disagreement rather than agreement with items expressing beliefs in brilliance. An ANOVA with department and gender as factors yielded two statistically significant main effects (department: *F*_(4,1281)_ = 4.83; *p* = 0.001; gender: *F*_(1,1281)_ = 6.97; *p* = 0.01], but no statistically significant interaction [*F*_(4,1281)_ = 1.46; *p* = 0.21]. Thus, regardless of their gender, students from different departments differed systematically in their endorsement of brilliance as a necessary precondition for success. Descriptively, Engineering and Computer Science students reported lower beliefs in brilliance than students of Physics or Mathematics. The effect size for the difference between the lowest and highest scoring departments (Mechanical Engineering vs. Physics) is *d* = 0.35. Across departments, women were more likely than men to endorse raw talent as a necessary condition for academic success in their chosen field of study (*d* = 0.22).

**Table 1:** Belief in brilliance (min. 1, max. 7) according to subject and gender.

	Male students			Female students			All students		
	***M***	**SD**	***n***	***M***	**SD**	***n***	***M***	**SD**	***n***

Mathematics	3.79	1.21	140	4.10	0.97	48	3.87	1.16	188
Physics	3.87	1.20	161	3.95	1.06	54	3.89	1.16	215
Comp. Sci.	3.53	1.26	234	4.07	1.24	37	3.60	1.27	271
Electric. Eng.	3.50	1.11	198	3.71	1.12	39	3.53	1.11	237
Mech. Eng.	3.52	1.04	324	3.48	0.89	56	3.51	1.02	380
Total	3.61	1.16	1057	3.85	1.06	234	3.65	1.14	1291

### Belonging Uncertainty

In our sample, the internal consistency of the three BU items was very low (Cronbach’s α = 0.42), which was due to one of the items (“When things are going well, I feel that I really belong at ETH”) not loading with the other two. This is an issue already discussed by the authors of the original scale, who report that this items loads with the other two in some samples but not in others (Walton, 2018).

Thus, we dropped the item and used only the mean of the two remaining items for our sample (Cronbach’s α = 0.82). Table 2 gives the BU score for students of the five department and for both genders. Higher values indicate greater belonging uncertainty (the answering scale runs from 1 to 7, with 4 representing the middle of the scale). Most mean values are in the upper half of the scale, indicating agreement rather than disagreement with the belonging uncertainty items.

**Table 2:** Belonging Uncertainty (min. 1, max. 7) according to subject and gender.

	Male students			Female students			All students		
	***M***	**SD**	***n***	***M***	**SD**	***n***	***M***	**SD**	***n***

Mathematics	4.13	1.92	140	5.28	1.51	48	4.42	1.89	188
Physics	4.78	1.90	161	5.19	1.76	54	4.89	1.87	215
Comp. Sci.	4.51	1.77	234	5.23	1.71	37	4.61	1.78	271
Electric. Eng.	4.41	1.77	198	5.12	1.97	39	4.53	1.82	237
Mech. Eng.	3.94	1.75	324	4.74	1.63	56	4.05	1.75	380
Total	4.31	1.83	1057	5.10	1.71	234	4.45	1.83	1291

An ANOVA with department and gender as factors yielded two statistically significant main effects [department: *F*_(4,1281)_ = 3.33; *p* = 0.01; gender: *F*_(1,1281)_ = 33.22; *p* < 0.001], but no statistically significant interaction [*F*_(4,1281)_ = 0.84; *p* = 0.49]. Across departments, women reported higher levels of belonging uncertainty than men did (*d* = 0.45). Comparing departments, Mechanical Engineering students reported the lowest level of belonging uncertainty, and Physics students the highest, with the other departments somewhere in between. The effect size for the difference between the lowest and highest scoring departments (Mechanical Engineering vs. Physics) is *d* = 0.46.

### Correlation and Mediation Analyses

The two scales, FSAB and BU, showed a small, positive correlation (*r* = 0.19; *p* < 0.001; *n* = 1291) that was very similar in size for men (*r* = 0.19; p < 0.001; *n* = 1057) and women (*r* = 0.18; *p* = 0.01; *n* = 234).

Further, the results were similar for all departments, with r ranging between *r* = 0.16 (Electrical Engineering) and *r* = 0.21 (Mechanical Engineering).

In a mediation analysis (using the PROCESS macro for SPSS as described by Hayes, 2017), we explored the possibility whether women’s higher belief in brilliance might explain their higher belonging uncertainty. The total effect of gender on belonging uncertainty (*B* = 0.79; *p* < 0.001) could be split into a direct effect (i.e., unmediated; *B* = 0.72; *p* < 0.001), and an indirect effect (i.e., mediated by belief in brilliance; *B* = 0.07, bootstrapped 95% CI: [0.02; 0.13]). Thus, a relatively small, but significant portion of the effect of gender on belonging uncertainty could be explained by women’s higher belief in brilliance.

**FIGURE 1:**
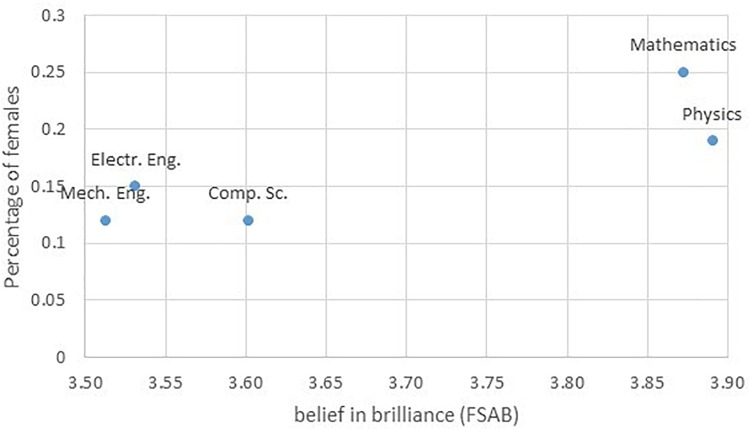
Scatterplot of belief in brilliance and the percentage of female students in the five departments.

Figure 1 shows a scatterplot of students’ endorsement of brilliance against the percentage of female students in their field. A negative trend would have been expected in analogy to the negative correlation between faculty’s endorsement of brilliance and the percentage of female PhD recipients in their field found by Leslie et al. (2015). We only have five departments to plot, and thus cannot reliably calculate a statistical correlation. However, the direction of the association appears to be positive rather than negative, with the two departments with the highest percentage of female students (Physics and Mathematics) also being those with the highest general endorsement of brilliance as a prerequisite for success. Thus, at least in our sample, it is math-intensiveness, rather than the minority status of women, which is associated with higher belief in brilliance of students in the respective field.

## Discussion

This is the first study testing the field-specific ability beliefs (using the instrument developed by Leslie et al., 2015) of university students in the respective fields. In the following, we compare our results to the previous findings by Leslie and colleagues in United States samples of academics (Leslie et al., 2015) and lay people (Meyer et al., 2015). Further, we discuss the correlation between field-specific ability beliefs emphasizing brilliance (belief in brilliance) and belonging uncertainty that we found in our sample.

### Comparison With Previous Findings by Leslie et al. (2015)

In line with the previous studies by Leslie and colleagues (Leslie et al., 2015; Meyer et al., 2015), we find that academic fields differ with respect to the amount of “brilliance” that is assumed to be required for success. Within STEM fields, we find the highest endorsement of brilliance-related statements in the fields of Physics and Mathematics, which replicates the findings by Leslie and colleagues (Leslie et al., 2015; Meyer et al., 2015). In our sample, students in nearly all fields and of both genders were more likely to reject, rather than endorse, brilliance-related statements. In contrast, the mean values reported by Leslie et al. (2015) for faculty members were all above the midpoint of their answering scale, indicating agreement rather than rejection. Thus, overall, the belief in brilliance was substantially lower in our student sample than in Leslie et al’s. (2015) faculty sample. This might have been due, first if all, to different operationalizations of success in our student vs. Leslie et al’s. (2015) faculty sample: While we used the term “success” in a rather unspecific way [e.g., “Being successful in my subject of study requires a special aptitude (…)”], Leslie et al. (2015) specifically asked their faculty respondents what it would take to be a top scholar in a given field [e.g., “Being a top scholar in my subject of study requires a special aptitude (…)”]. It is possible that we would have found higher agreement rates if we had framed success in this way to our students as well, or that Leslie et al. (2015) would have found lower agreement rates if they had asked faculty members about factors influencing success as a student of their field more generally. This is, however, a question that only additional research can answer. On the other hand, the different endorsement rates could also reflect the different levels of seniority and professional status of the respondents: Given the human tendency for self-enhancing attributions of success (Miller and Ross, 1975), the successful academics surveyed by Leslie et al. (2015) should have been more willing to attribute success to talent or “brilliance” than the first-year students surveyed in our study. Further, to the extent that emphasis on “brilliance” is part of the culture of a given field that experts acquire during the course of their studies, belief in brilliance would be expected to be stronger in field experts (i.e., faculty) than novices (i.e., first-year students). Finally, the difference in our findings to those of Leslie et al. (2015) might also be due to more general cultural differences between the United States and Switzerland (or in a broader sense Europe).

Meyer et al. (2015) studied a United States sample of lay people with varying degrees of exposure to the various fields of science. They, too, found that their respondents predominantly endorsed brilliance-related statements (with means for the majority of all surveyed fields, and for all STEM fields, above the scale mid-point). Thus, the belief in the necessity of innate talent for success in academic fields might be a more typical belief in United States than in Swiss samples.

Our data set allowed us to disentangle “math intensiveness” and “minority status of women”; we found that the highest belief in brilliance was found in the most math-intensive fields, rather than in those fields with the fewest numbers of women. In contrast, previous research with faculty members (Leslie et al., 2015) as well as with lay people (Meyer et al., 2015) found a (negative) correlation between an emphasis of brilliance for success in a given field and the percentage of women among its successful graduates (at Ph.D. level). Our study was not a direct replication of the Leslie et al. (2015) study, as it used a different respondent sample (first-year students instead of faculty) and a different criterion (percentage of females among enrolled students, and not among successful graduates). Further, we only studied a very restricted sub-set of five STEM fields. Nevertheless, our findings demonstrate that the correlation found by Leslie et al. (2015) is only observable when considering the full range of academic subjects. Within STEM, math-intensiveness may be a better predictor of belief in brilliance.

Finally, in contrast to previous studies, we found systematic gender differences in field-specific ability beliefs, with women being less reluctant than men to endorse brilliance-related statements. We also found higher levels of belonging uncertainty in women than in men, as well as a positive correlation between belief in brilliance and belonging uncertainty for both genders. These findings will be discussed in detail in the next section.

### Belief in Brilliance and Belonging Uncertainty

To the best of our knowledge, our study is the first showing that the more students believe that innate talent is a prerequisite for success in their field of study, the more likely they are to experience belonging uncertainty. This means that they are less likely to think that they actually do belong in their chosen field. The correlation was small (around *r* = 0.19 for both genders), which would be expected given the many possible factors that could influence students’ perception of “belonging” to their chosen field of study. Nevertheless, we consider our result relevant, as belonging uncertainty is a plausible mechanism by which belief in brilliance could influence the paths that men and women chose for their future careers inside or outside of STEM fields.

Correlation does not imply causation. On the one hand it may be that the more students believe that success in their field depends on raw talent, the more anxious they feel about the amount of talent that they actually possess and the less certain they are that it will suffice to succeed in their studies, resulting in increased belonging uncertainty. The belief that academic success depends on talent, which cannot be increased and thus is largely out of one’s control, corresponds to a “fixed,” or entity theory of talent (cf. Dweck, 2007; Yeager and Dweck, 2012). Thus, the correlation between belief in brilliance and belonging uncertainty may have been mediated by students’ belief in an entity theory of talent.

On the other hand, students experiencing failures (e.g., trouble keeping up with coursework), or negative stereotypes (e.g., “women are not smart enough to succeed in this field”) early in their course of study may develop the hypotheses that (a) their chosen field requires prerequisite talent out of their reach, and therefore (b) is not be the field in which they actually belong. Thus, students searching explanations for their failures and struggles may have developed both stronger beliefs in brilliance and stronger belonging uncertainty.

While the magnitude of the correlation between students’ beliefs in brilliance and their belonging uncertainty was similar for both genders, women reported higher levels of both variables than men did. As the score assessing belief in brilliance included several reverse coded items, this finding is unlikely to merely reflect a tendency of women choosing more affirmative answers than men. Also, the gender difference was found in 4 out of 5 of the surveyed departments (see Table 1). Thus, it cannot result from the overrepresentation of students from fields with a high emphasis on brilliance in our female sample. According to the two explanations developed in the last paragraph, we cannot exclude the possibility that the women in our sample had a more “fixed” theory of talent *a priori*. This may have made them more anxious about their own talent, and thus they were less certain to belong to their chosen field.

On the other hand, women may also have experienced more failures during their first weeks of study than men did, leading them to report higher levels of belonging uncertainty, and making them more likely to assume that their chosen field requires levels of innate talent of which they do not dispose. Finally, experiencing failure and/or bias may have led women to activate negative stereotypes about their gender. Consequently, they may have developed the belief that they lack essential talents for being successful in their chosen field because of their gender. Therefore, they may have been less likely to reject brilliance-related statements than men are, and more likely to experience belonging uncertainty (cf. Walton and Cohen, 2007). However, no conclusive inferences concerning the reason for women’s higher belonging uncertainty and higher beliefs in brilliance can be drawn based on the obtained data. In future studies, in addition to assessing field-specific ability beliefs and belonging uncertainty, it would therefore be interesting to assess students’ implicit stereotypes about science and gender, their goal orientations and attribution patterns, and their experiences of successes, failures, and obstacles (including negative stereotypes) during their studies.

### Field-Specific Ability Beliefs and Gendered Paths Into STEM

In line with the findings obtained by Leslie and colleagues (Leslie et al., 2015; Meyer et al., 2015), our results show that STEM fields differ in the amount of “brilliance” that people assume to be required for success. From our surveys we cannot conclude to what extent these beliefs reflect true affordances of specific fields. However, a study run with an earlier cohort from the same university revealed that general intelligence could better explain achievement differences in Mathematics exams among students from Physics and Mathematics than among students from Mechanical Engineering (Berkowitz and Stern, 2018). These data suggest that field-specific ability profiles are reflected in field-specific ability beliefs, which themselves may shape processes of evaluation and selection information on who becomes a successful scholar in a given field. Field-specific ability beliefs emphasizing the necessity of brilliance, combined with the cultural stereotype of associating brilliance with men rather than women, will lead to practices and processes in a field that eventually exclude women (Leslie et al., 2015) and may undermine women’s interest in specific fields (Bian et al., 2018). Our results show that, even among young women who have chosen to study a STEM subject, biases linking science to “brilliance” are prevalent, and can partly explain their higher belonging uncertainty in these fields.

## Ethics Statement

This study was carried out in accordance with the Guidelines for Research Integrity and Good Scientific Practice by the Ethics Commission at the ETH Zurich. All subjects gave written informed consent. The protocol was approved by the Ethics Commission of the ETH Zurich.

## Author Contributions

All authors made substantial contributions to the conception and design of the study. ES and RS recruited the support of the participating departments, made substantial contributions to interpreting the results, and to revising several drafts of the manuscript. AD devised the materials, supervised data collection, analyzed the data, and prepared the manuscript, tables, and figure.

## Conflict of Interest Statement

The authors declare that the research was conducted in the absence of any commercial or financial relationships that could be construed as a potential conflict of interest.

## References

[B1] Berkowitz M. Stern E. (2018). Which cognitive abilities make the difference? Predicting academic achievements in advanced STEM studies. *J. Intell.* 6 1–24. 10.3390/jintelligence6040048 PMC6480791 31162475

[B2] Berkowitz M. Stern E. Hofer S. Deiglmayr A. (in press). “Girls, boys and schools: On gender (in)equalities in education,” in *The Cambridge International Handbook on Psychology of Women*, eds Cheung F. M. Halpern D. F. (Cambridge: Cambridge University Press).

[B3] Bian L. Leslie S. J. Murphy M. Cimpian A. (2018). Messages about brilliance undermine women’s interest in educational and professional opportunities. *J. Exp. Soc. Psychol.* 76 404–420. 10.1016/j.jesp.2017.11.006

[B4] Ceci S. J. Ginther D. K. Kahn S. Williams W. M. (2014). Women in academic science: a changing landscape. *Psychol. Sci. Public Int.* 15 75–141. 10.1177/1529100614541236 26172066

[B5] Cheryan S. Ziegler S. A. Montoya A. K. Jiang L. (2017). Why are some STEM fields more gender balanced than others? *Psychol. Bull.* 143 1–35. 10.1037/bul0000052 27732018

[B6] Dweck C. (2007). “Is math a gift? Beliefs that put females at risk,” in *Why aren’t more Women in 1460 Science? Top Researchers Debate the Evidence*, eds Ceci S. J. Williams W. M. (Washington, WA: APA Press), 10.1037/11546-004

[B7] Hayes A. (2017). *Introduction to Mediation, Moderation, And Conditional Process Analysis*, 2nd Edn. New York, NY: Guilford.

[B8] Hofer S. I. (2015). Studying gender bias in physics grading: the role of teaching experience and country. *Int. J Sci. Edu.* 37 2879–2905. 10.1080/09500693.2015.1114190

[B9] Leslie S.-J. Cimpian A. Meyer M. Freeland E. (2015). Expectations of brilliance underlie gender distributions across academic disciplines. *Science* 347 262–265. 10.1126/science.1261375 25593183

[B10] Meyer M. Cimpian A. Leslie S. J. (2015). Women are underrepresented in fields where success is believed to require brilliance. *Front. Psychol.* 6:235. 10.3389/fpsyg.2015.00235 25814964 PMC4356003

[B11] Miller D. I. Eagly A. H. Linn M. C. (2015). Women’s representation in science predicts national gender-science stereotypes: evidence from 66 nations. *J. Edu. Psychol.* 107 631–644. 10.1037/edu0000005

[B12] Miller D. Ross M. (1975). Self-serving biases in the attribution of causality: fact or fiction? *Psychol. Bull.* 82 213–225. 10.1037/h0076486

[B13] Moss-Racusin C. A. Dovidio J. F. Brescoll V. L. Graham M. Handelsman J. (2012). Science faculty’s subtle gender biases favor male students. *Proc. Natl. Acad. Sci.* 109 16474–16479. 10.1073/pnas.1211286109 22988126 PMC3478626

[B14] Nosek B. A. Banaji M. R. Greenwald A. G. (2002). Math = male, me = female, therefore math ≠ me. *J. Pers. Soc. Psychol.* 83 44–59. 10.1037/0022-3514.83.1.44 12088131

[B15] Proudfoot D. Kay A. C. Koval C. Z. (2015). A gender bias in the attribution of creativity: archival and experimental evidence for the perceived association between masculinity and creative thinking. *Psychol. Sci.* 96 1751–1761. 10.1177/0956797615598739 26386015

[B16] Stoet G. Geary D. C. (2018). The gender-equality paradox in Science. Technology, Engineering, and Mathematics education. *Psychol. Sci.* 29 581–593. 10.1177/0956797617741719 29442575

[B17] Thébaud S. Charles M. (2018). Segregation, stereotypes, and STEM. *Soc. Sci.* 7 1–18.

[B18] Verniers C. Martinot D. (2015). Perception of students’ intelligence malleability and potential for future success: unfavourable beliefs towards girls. *Br. J Educ. Psychol.* 85 289–299. 10.1111/bjep.12073 25817078

[B19] Walton G. M. (2018). *Belonging and Belonging Uncertainty. Online Ressource.* http://gregorywalton-stanford.weebly.com/uploads/4/9/4/4/49448111/belonging_belonginguncertainty.pdf (accessed October 30 2018).

[B20] Walton G. M. Cohen G. L. (2007). A question of belonging: race, social fit, and achievement. *J. Pers. Soc. Psychol.* 92 82–96. 10.1037/0022-3514.92.1.82 17201544

[B21] Walton G. M. Cohen G. L. (2011). A brief social-belonging intervention improves academic and health outcomes of minority students. *Science* 331 1447–1451. 10.1126/science.1198364 21415354

[B22] Walton G. M. Logel C. Peach J. M. Spencer S. J. Zanna M. P. (2015). Two brief interventions to mitigate a ”chilly climate” transform women’s experience, relationships, and achievement in engineering. *J. Edu. Psychol.* 107 468–485. 10.1037/a0037461

[B23] Wang M. T. Degol J. L. (2017). Gender gap in Science, Technology, Engineering, and Mathematics (STEM): current knowledge, implications for practice, policy, and future directions. *Edu. Psychol. Rev.* 29 119–140. 10.1007/s10648-015-9355-x 28458499 PMC5404748

[B24] Yeager D. S. Dweck C. S. (2012). Mindsets that promote resilience: when students believe that personal characteristics can be developed. *Educ. Psychol.* 47 302–314. 10.1080/00461520.2012.722805

