# Supplementary material for: Beliefs in “Brilliance” and Belonging Uncertainty in Male and Female STEM Students
Source: Front Psychol. 2019 May 28;10:1114. doi: 10.3389/fpsyg.2019.01114 (PMC6546818; doi:10.3389/fpsyg.2019.01114)
Supplement: Supplementary file 1 [file Table_1.docx]

**Supplementary Table 1:** Survey items. The original items of the two scales field-specific ability beliefs (FSAB, Leslie et al., 2015) and belonging uncertainty (BU; Walton & Cohen, 2007) were translated to German, adapted to the surveyed sample, and presented in an intermixed fashion.

|  | Item as seen by the students | English re-translation | Source |
| --- | --- | --- | --- |
| 1 | In meinem Studienfach sind die wichtigsten Erfolgsfaktoren Motivation und Durchhaltevermögen; die reine Begabung ist zweitrangig. | In my subject of study, the most important factors for success are motivation and sustained effort; raw ability is secondary. (reverse coded) | FSAB (Leslie et al., 2015) |
| 2 | Manchmal habe ich das Gefühl, dass ich an die ETH gehöre, und manchmal nicht. | Sometimes I feel that I belong at ETH, and sometimes I don’t. | BU (Walton & Cohen, 2007) |
| 3 | Um in meinem Studienfach erfolgreich zu sein, muss man von vorn herein eine spezifische Begabung mitbringen, die sich nicht erlernen lässt. | Being successful in my subject of study requires a special aptitude that just can’t be learned. | FSAB (Leslie et al., 2015) |
| 4 | Wenn es gerade schlecht läuft, habe ich das Gefühl, dass ich vielleicht doch nicht an die ETH gehöre. | When things are going badly, I feel that maybe I don’t belong at ETH after all. | BU (Walton & Cohen, 2007) |
| 5 | In meinem Studienfach kann im Prinzip jeder erfolgreich sein, wenn sie / er sich hinreichend anstrengt. | In principle, anyone can be successful in my subject of study if they work hard enough. (reverse coded) | FSAB (Leslie et al., 2015) |
| 6 | Wenn es gerade gut läuft, habe ich das Gefühl, dass ich wirklich an die ETH gehöre. | When things are going well, I feel that I really belong at ETH. | BU (Walton & Cohen, 2007) |
| 7 | Wenn man in meinem Studienfach Erfolg haben will, ist harte Arbeit alleine nicht ausreichend; man braucht zusätzlich eine angeborene Begabung bzw. ein bestimmtes Talent. | If you want to succeed in my subject of study, hard work alone is not sufficient; in addition, you need to have an innate gift or talent. | FSAB (Leslie et al., 2015) |
